# Treatment of Terminal Peritoneal Carcinomatosis by a Transducible p53-Activating Peptide

**DOI:** 10.1371/journal.pbio.0020036

**Published:** 2004-02-17

**Authors:** Eric L Snyder, Bryan R Meade, Cheryl C Saenz, Steven F Dowdy

**Affiliations:** **1**Howard Hughes Medical Institute, Chevy ChaseMarylandUnited States of America; **2**Washington University School of Medicine, St. LouisMissouriUnited States of America; **3**Department of Cellular and Molecular Medicine, School of MedicineUniversity of California, San Diego, La Jolla, CaliforniaUnited States of America; **4**Department of Reproductive Medicine, School of MedicineUniversity of California, San Diego, La Jolla, CaliforniaUnited States of America

## Abstract

Advanced-stage peritoneal carcinomatosis is resistant to current chemotherapy treatment and, in the case of metastatic ovarian cancer, results in a devastating 15%–20% survival rate. Therapeutics that restore genes inactivated during oncogenesis are predicted to be more potent and specific than current therapies. Experiments with viral vectors have demonstrated the theoretical utility of expressing the *p53* tumor suppressor gene in cancer cells. However, clinically useful alternative approaches for introducing p53 activity into cancer cells are clearly needed. It has been hypothesized that direct reactivation of endogenous p53 protein in cancer cells will be therapeutically beneficial, but few tests of this hypothesis have been carried out in vivo. We report that a transducible D-isomer RI-TATp53C′ peptide activates the p53 protein in cancer cells, but not normal cells. RI-TATp53C′ peptide treatment of preclinical terminal peritoneal carcinomatosis and peritoneal lymphoma models results in significant increases in lifespan (greater than 6-fold) and the generation of disease-free animals. These proof-of-concept observations show that specific activation of endogenous p53 activity by a macromolecular agent is therapeutically effective in preclinical models of terminal human malignancy. Our results suggest that TAT-mediated transduction may be a useful strategy for the therapeutic delivery of large tumor suppressor molecules to malignant cells in vivo.

## Abstract 2

Specific activiation of the tumor suppressor protein p53, using a transducible p53 C-terminal peptide, dramatically increases survival in a mouse model of peritoneal carcinomatosis. This peptide offers therapeutic potential for tumors in which p53 is mutated

## Introduction

Most patients who succumb to cancer do so not from primary tumor burden, but from metastatic disease (Fidler 2003). For example, advanced-stage peritoneal carcinomatosis (e.g., from metastatic ovarian and breast cancer) and disseminated peritoneal lymphomas are often resistant to current chemotherapy treatment (Parsons et al. 1996). Posttreatment survival rates for patients presenting with metastatic ovarian peritoneal carcinomatosis or lymphoma are less than 20% and less than 50%, respectively (Lam and Zhao 1997; Deppe and Baumann 2000; Hofstra et al. 2000). Consequently, the development of novel therapeutic strategies to reverse these numbers is clearly warranted.

A significant effort has been aimed at understanding the function of tumor suppressor gene pathways that are genetically and epigenetically altered during oncogenesis (Macleod 2000). One rationale for the study of tumor suppressor pathways is the hypothesis that reconstitution of these pathways in cancer patients will be therapeutically beneficial (Macleod 2000). The p53 tumor suppressor protein induces growth arrest and apoptosis in response to cellular stress (Vousden and Lu 2002). Mutation of genes in the p53 pathway is thought to be nearly universal in human cancer (Vousden and Lu 2002). Thus, any strategy designed to restore p53 activity in tumor cells will likely be an effective means of inducing cancer cell death and will be applicable to a large fraction of cancer patients. The inability of large tumor suppressor proteins, all of which are intracellular, to cross the plasma membrane precludes the therapeutic administration of recombinant tumor suppressors in a manner analogous to administration of extracellular biological therapeutics (e.g., insulin or G-CSF). Thus, the development of an efficient methodology for restoring tumor suppressor function to cancer cells in vivo remains a challenge for both basic and clinical researchers.

Gene therapy approaches aimed at restoring tumor suppressor function have been extensively investigated. Both viral and nonviral vectors have been employed to express exogenous tumor suppressor genes, such as *p53*, in cancer cells (McCormick 2001). Although gene therapy may be useful under certain conditions, problems associated with immunogenicity and lack of systemic biodistribution to disseminated metastases are likely to curtail its anticancer efficacy (McCormick 2001).

Delivery of macromolecules by protein transduction has recently emerged as an alternative methodology for directly introducing tumor suppressor proteins into cancer cells in vivo. Several small cationic peptides, including TAT, Antp, and polyArg (referred to as protein transduction domains [PTDs]), are capable of traversing the plasma membrane and entering the cytoplasm of cells by a concentration-dependent, but receptor-independent, macropinocytic mechanism (Wadia et al. 2004). PTDs have recently been used to deliver a wide range of cargo, including biologically active proteins, peptides, nucleic acids, and iron beads, into cells in culture (Fischer et al. 2001; Lindsay 2002). PTDs have also been employed to deliver biologically active cargo into most, if not all, tissues in preclinical models (Schwarze et al. 1999).

Because of the presence of either wild-type or mutant p53 protein in most tumors, it has been hypothesized that restoration of endogenous p53 activity in cancer cells will be a therapeutically efficacious alternative to delivery of exogenous p53. However, this hypothesis has been tested in vivo in only a limited number of cases (Foster et al. 1999; Bykov et al. 2002) and has never been tested in preclinical models of terminal human malignancy. We therefore focused on a strategy to activate endogenous p53 in cancer cells by PTD-mediated delivery. The C-terminus of p53 is a lysine-rich domain that is subjected to a variety of posttranslational modifications (Apella and Anderson 2001). A peptide derived from the C-terminus was previously shown by D. Lane's group (University of Dundee, United Kingdom) to activate specific DNA binding by p53 in vitro by an unknown mechanism (Hupp et al. 1995). In cancer cells, p53C′ peptide can induce apoptosis by activating wild-type p53 protein and by restoring function to several p53 DNA contact mutants. Importantly, the p53C′ peptide also restores specific DNA binding to some p53 DNA contact mutants in vitro and induces apoptosis in cancer cells expressing p53 DNA contact mutants (Selivanova et al. 1997, 1998; Kim et al. 1999). However, the peptide fails to induce apoptosis in p53-deficient tumor cells or in tumor cells containing *p53* structural mutations. In contrast, primary cells are resistant to p53C′ peptide action (Selivanova et al. 1997; Kim et al. 1999). This resistance is likely a result of the extremely low levels of endogenous p53 present in normal cells and the absence of continual DNA damage often associated with tumor cells (Selivanova et al. 1997; Kim et al. 1999).

Here we report a proof-of-concept that in vivo delivery of a transducible, proteolytically stable p53C′ peptide (termed RI-TATp53C′) is a therapeutically effective means of activating the p53 tumor suppressor pathway in preclinical models of terminal metastatic cancer.

## Results

### Activation of p53 by Transducible Retro-Inverso D-Isomer p53C′ Peptide

**Figure 1:**
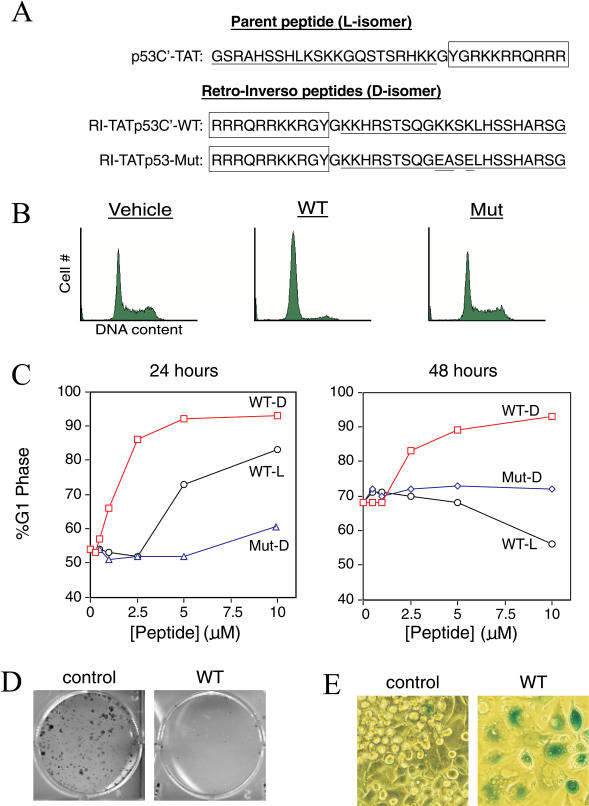
RI-TATp53C′ Induces the Hallmarks of p53 Activity in TA3/St Mammary Carcinoma Cells (A) Sequence of p53C′TAT peptide (L-amino acids) and its retro-inverso analogue (D-amino acids). To generate a negative control peptide, three essential lysine residues (Selivanova et al. 1997) were mutated while leaving the remaining peptide sequence intact. (B) Induction of G1 arrest in TA3/St cells by wild-type RI-TATp53C′, but not mutant peptide, 24 h after peptide addition. (C) Dose-dependent induction of G1 arrest by RI-TATp53C′ (open square) (D-amino acids) and the less potent p53C′TAT (open circle) (L-amino acids) but not mutant (open triangle) peptide at 24 h (left) and 48 h (right) after single treatment. (D) Induction of a permanent growth arrest in TA3/St cells by RI-TATp53C′. Cells were treated with RI-TATp53C′ peptide or vehicle for 2 d, replated, and allowed to proliferate in the presence of serum for 10 d. Colonies were then stained with methylene blue. (E) Induction of a senescence-like phenotype in TA3/St cells by RI-TATp53C′. Cells were treated with RI-TATp53C′ peptide and stained for acidic β-galactosidase activity.

Although PTDs solve one major obstacle to the use of intracellular peptides as therapeutics, the susceptibility of peptides to degradation in vivo remains problematic. To circumvent the problem of proteolytic degradation, we synthesized a retro-inverso version of the parental p53C′ peptide by inverting the peptide sequence, using D-isomer residues, and adding the TAT PTD to obtain a transducible RI-TATp53C′ peptide (Figure 1A). This double inversion of peptide structure often leaves the surface topology of the sidechains intact and has been used extensively to stabilize biologically active peptides for in vivo applications (Chorev and Goodman 1993). Because of their greater stability, retro-inverso peptides often display increased potency.

To determine whether the RI-TATp53C′ peptide retained functionality, we compared the transducible parental L-isomer p53C′TAT and D-isomer RI-TATp53C′ peptides for the ability to induce a cell cycle arrest (Figure 1B). Treatment of murine TA3/St mammary carcinoma cells (which express wild-type p53) with either the L-isomer p53C′TAT or D-isomer RI-TATp53C′ peptides resulted in a concentration-dependent G1 cell cycle arrest (Figure 1B and 1C). The control D-isomer mutant peptide had little to no effect (Figure 1B and 1C). Compared to the L-isomer p53C′TAT, the D-isomer RI-TATp53C′ peptide induced a stronger cell cycle arrest at substantially lower concentrations (Figure 1C). A single administration of the L-isomer p53C′TAT peptide partially arrested cells for 24 h, but by 48 h cells had reentered the cell cycle (Figure 1C). In contrast, a single dose of the D-isomer RI-TATp53C′ peptide was sufficient to sustain a G1 arrest for greater than 7 d (Figure 1C; data not shown).

To ascertain whether sustained arrest required the continuous presence of RI-TATp53C′ peptide, TA3/St cells were treated with peptide or vehicle for 2 d and then replated under mitogenic conditions in the absence of peptide. Peptide-treated tumor cells formed less than 1% as many colonies as vehicle-treated cells (Figure 1D). This observation suggested that RI-TATp53C′ peptide induced a permanent growth arrest in TA3/St cells. We therefore assayed RI-TATp53C′ peptide-treated cells for induction of senescence, a state of terminal arrest that can be induced by p53 activation (Roninson et al. 2002). By 6 d after peptide addition, greater than 80% of viable TA3/St cells were positive for acidic β-galactosidase activity (Figure 1E), the standard marker of senescence (Roninson et al. 2002). The treated cells also displayed other features of senescence (Roninson et al. 2002), including increased size, increased granularity, and a flattened morphology (Figure 1E; data not shown). These observations suggest that treatment of mammary carcinoma cells with the RI-TATp53C′ peptide induces hallmarks of p53 activation, namely a G1 cell cycle arrest followed by induction of senescence.

**Figure 2:**
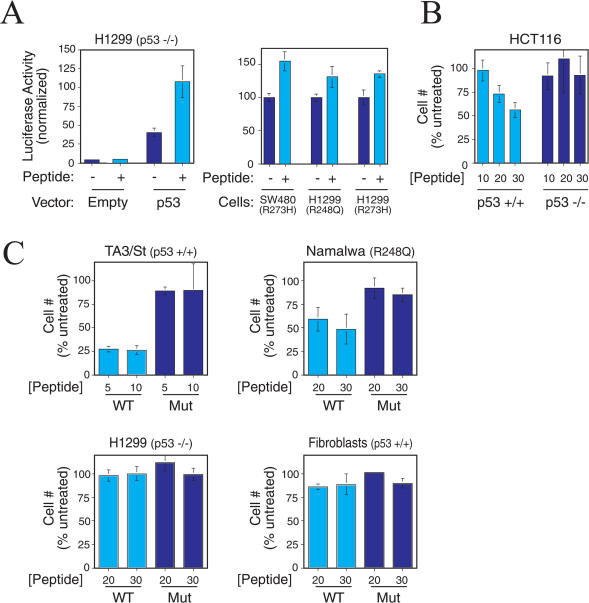
RI-TATp53C′ Peptide Activates p53-Dependent Transcription and Inhibits Tumor Cells Expressing p53 (A, left) Induction of transcription from a p53-dependent promoter by RI-TATp53C′ only when p53 protein is expressed. H1299 cells (*p53^−/−^*) were cotransfected with p53-responsive reporter (PG13-Luc) and either empty vector or p53 expression vector. Depicted are mean and standard deviation of triplicate results that are representative of multiple experiments. (A, right) RI-TATp53C′ peptide activates p53-dependent transcription in cells expressing DNA contact mutant p53. SW480 cells containing a DNA contact mutant (R273H) p53 were transfected with p53-dependent reporter (PG13-Luc). H1299 cells (*p53* ^−/−^) were co-transfected with PG13-Luc and either R248Q or R273H mutant p53 expression vector. RI-TATp53C′ was added to cells, and promoter activity was assessed 24 h later. (B) Inhibition of tumor cell proliferation in a p53-dependent manner by RI-TATp53C′. Increasing concentrations of peptide were added to HCT 116 cells (*p53^+/+^*) and their *p53^−/−^* isogenic derivatives. After 2 d, the number of viable cells was assessed by Trypan blue exclusion and normalized to the number of viable untreated cells. Mean and standard deviation of multiple experiments are depicted. (C) Inhibition of the proliferation of tumor cells expressing wild-type or mutant p53, but not *p53^−/−^* tumor cells or nontransformed human fibroblasts. Cell viability was assessed as in (B). Mean and standard deviation of multiple experiments are depicted.

We next investigated the ability of the RI-TATp53C′ peptide to transcriptionally activate p53-responsive genes. We transiently transfected p53 null human H1299 lung carcinoma cells with p53-dependent luciferase reporter plasmid (PG13-Luc) and either wild-type p53 expression plasmid or empty vector. The use of p53 null cells allows for negative controls that are not possible in cells expressing endogenous p53. As expected, we observed a p53-dependent induction of luciferase activity in cells transfected with p53 expression vector (Figure 2A). However, RI-TATp53C′ peptide treatment of cells transfected with p53 expression vector resulted in a significant increase in p53-dependent luciferase activity (Figure 2A, left). Consistent with observations in TA3/St cells (see Figure 1B and 1C), mutant peptide and L-isomer p53C′TAT displayed substantially reduced potency in this assay when compared to RI-TATp53C′ peptide (data not shown). Importantly, RI-TATp53C′ peptide treatment of cells transfected with empty vector and luciferase plasmids caused no increase in p53 target promoter activity (Figure 2A). In addition, RI-TATp53C′ peptide activated p53-dependent transcription in SW480 colon carcinoma cells expressing a p53 DNA contact mutant (R273H) and in H1299 p53 null colon carcinoma cells transfected with a p53 DNA contact mutant (R248Q and R273H) (Figure 2A, right), though to a lesser extent than in the presence of wild-type p53 (Figure 2A, left). These observations show that RI-TATp53C′ peptide retains the ability to specifically activate p53-dependent gene transcription.

To confirm that the RI-TATp53C′ peptide inhibited tumor cell proliferation in a p53-dependent fashion, we compared parental *p53^+/+^* HCT116 colorectal carcinoma cells to HCT116 cells that were rendered p53-deficient at both loci by targeted genetic recombination (Bunz et al. 1998). Treatment of wild-type *p53* HCT116 cells with RI-TATp53C′ peptide inhibited cell proliferation in a dose-dependent manner (Figure 2B). In contrast, RI-TATp53C′ peptide treatment of p53-deficient HCT116 cells did not significantly alter the number of viable cells. p53-deficient human H1299 lung adenocarcinoma cells also failed to respond to the RI-TATp53C′ peptide (Figure 2C), further confirming the specificity of peptide action. RI-TATp53C′ peptide inhibited proliferation of TA3/St cells (*p53^+/+^*) and human Namalwa lymphoma cells that express a p53 hotspot DNA contact mutant (R248Q) (Figure 2C). In contrast, RI-TATp53C′ peptide did not alter the proliferation of normal human foreskin fibroblasts containing wild-type p53 (Figure 2C). These results are consistent with previous observations that certain p53 contact mutations are susceptible to p53C′ peptide activation and that the p53C′ peptide induces apoptosis in tumor cells, but not normal cells (Selivanova et al. 1997, 1998; Kim et al. 1999). Taken together, these observations demonstrate both the p53 and tumor dependency of the RI-TATp53C′ peptide.

### Systemic Delivery of RI-TATp53C′ Peptide Inhibits Solid Tumor Growth

**Figure 3:**
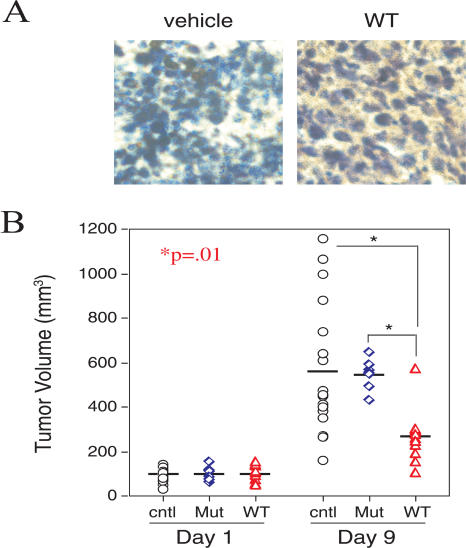
Solid Tumor Growth Is Inhibited by Systemic RI-TATp53C′ Peptide Administration (A) Delivery of RI-TATp53C′-biotin to subcutaneous TA3/St tumors after IP administration to immune competent A/J mice. (B) Reduction of solid TA3/St tumor growth in immune competent mice as a result of systemic administration of RI-TATp53C′. TA3/St cells were injected subcutaneously into A/J mice and allowed to grow to an average size of approximately 100 mm^3^. Mice were then sorted into treatment groups that received eight daily injections of vehicle (open circle) (*n* = 17), 650 μg of mutant peptide (open diamond) (*n* = 7), or 650 μg of wild-type RI-TATp53C′ peptide (open triangle) (*n* = 11). Final mean tumor volumes were 573 mm^3^ for vehicle-treated mice, 550 mm^3^ for mice treated with mutant peptide, and 268 mm^3^ for the wild-type RI-TATp53C′ peptide group.

We (Schwarze et al. 1999) and others (Datta et al. 2001; Harada et al. 2002) have previously shown that intraperitoneal (IP) administration of TAT–fusion peptides and proteins results in systemic delivery in animal models. Consistent with these observations, IP injection of a biotinylated RI-TATp53C′ peptide into mice harboring subcutaneous tumors resulted in distribution of the peptide throughout the tumor (Figure 3A).

We next tested the ability of IP administration of RI-TATp53C′ peptide to inhibit the growth of distant solid tumors in immune competent mice. Subcutaneous tumors in mice receiving either vehicle or mutant peptide grew rapidly, reaching an average volume of nearly 600 mm^3^ by the end of treatment (Figure 3B). In contrast, tumors in mice treated with wild-type RI-TATp53C′ peptide were significantly retarded in growth and reached a final mean volume less than 50% that of tumors in the control-treated mice (*p* = 0.01) (Figure 3B). These observations demonstrate that systemic delivery of RI-TATp53C′ peptide in immune competent mice can significantly inhibit the growth of an aggressively proliferating solid tumor at a distant site.

### RI-TATp53C′ Peptide Treatment of Terminal Peritoneal Carcinomatosis

Because of their encapsulation and ectopic site of growth, subcutaneous tumors fail to replicate many of the features of terminal human cancer. We therefore tested the efficacy of RI-TATp53C′ peptide in a terminal peritoneal carcinomatosis mouse model that more closely resembles metastatic human disease. TA3/St carcinoma cells inoculated into the peritoneum of immune competent, syngeneic A/Jax (A/J) mice proliferated in a rapid logarithmic fashion, doubling in 24 h and increasing their numbers 100-fold 5 d postinoculation (Nagy et al. 1993). This aggressive, terminal peritoneal carcinomatosis model of human disease has been used extensively to study the pathophysiology of peritoneal tumor growth (Nagy et al. 1993, Nagy et al. 1995).

**Figure 4:**
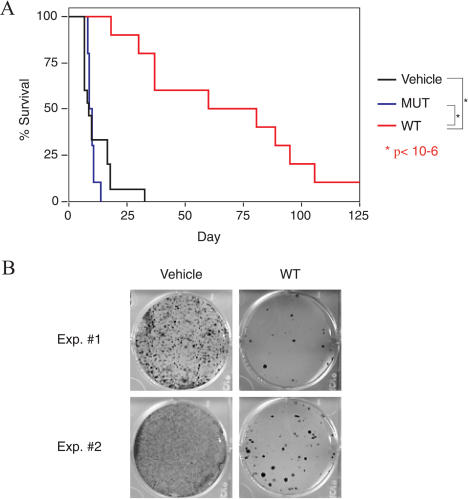
RI-TATp53C′ Treatment Extends Survival of Mice Harboring Terminal Peritoneal Carcinomatosis (A) A 6-fold increase in survival of A/J immune-competent mice harboring lethal TA3/St mammary peritoneal carcinomatosis burden after RI-TATp53C′ peptide treatment. A/J mice were given IP injections of TA3/St cells, and cells were allowed to double in number (approximately 24 h). Peritoneal tumor-bearing mice were then treated once a day for 12 consecutive days with vehicle (*n* = 15), 600 μg of wild-type RI-TATp53C′ (*n* = 10), or 600 μg of mutant peptide (*n* = 10). Mean survival duration was 11 d for vehicle-treated mice, 10 d for mice receiving mutant peptide, and greater than 70 d for the group receiving wild-type RI-TATp53C′ peptide. (B) Reduction of tumor cell number in vivo by RI-TATp53C′ treatment. Mice were injected with TA3/St tumor cells and treated with wild-type peptide as in (A). Three days after tumor cell injection, cells were flushed from the peritoneal cavity and serially diluted in 6-well plates. Growth of colonies was then assessed by methylene blue staining and used to measure the number of viable tumor cells present in the peritoneum after treatment with vehicle or wild-type peptide.

We assayed the ability of the RI-TATp53C′ peptide to alter the tumor burden and increase the longevity of mice harboring TA3/St peritoneal carcinomatosis. Vehicle-treated mice rapidly succumbed to peritoneal tumor burden with a mean survival time of 11 d (Figure 4A). Mice treated with control mutant peptide succumbed to their tumor burden with similar kinetics and a mean survival time of 10 d (Figure 4A). In contrast, peritoneal tumor-bearing mice treated with wild-type RI-TATp53C′ peptide lived on average more than 70 d after tumor inoculation (Figure 4A), a greater than 6-fold increase in lifespan over mutant peptide- or vehicle-treated mice (*p* < 10^−6^). These observations demonstrate the ability of transducible peptides to significantly extend survival in a mouse model of terminal peritoneal carcinomatosis.

We next investigated the biological consequences of peptide treatment to tumor cells in vivo. Peritoneal-TA3/St tumor-bearing mice were given daily injections of wild-type RI-TATp53C′ peptide or vehicle control. Mice were sacrificed 3 d after tumor cell inoculation for assessment of tumor burden. Vehicle-treated mice contained a significant tumor burden of recoverable dividing TA3/St tumor cells (Figure 4B). In contrast, mice treated with wild-type RI-TATp53C′ peptide showed a dramatic reduction in tumor cell number, suggesting that RI-TATp53C′ peptide treatment extended survival by directly inhibiting overall tumor proliferation. Consistent with cell culture studies, cell cycle analysis of tumor cells from peptide-treated mice showed an increase in G1 phase of the cell cycle (data not shown).

### RI-TATp53C′ Peptide Treatment of Terminal Peritoneal Lymphoma

**Figure 5:**
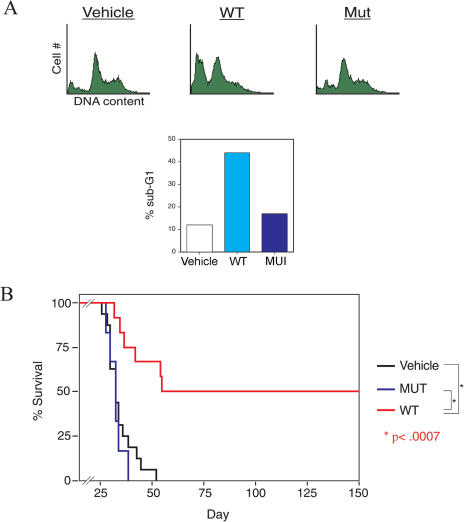
RI-TATp53C′ Treatment Leads to 50% Long-Term Survival of Mice Bearing Terminal Peritoneal Lymphoma (A) Treatment of human Namalwa B-cell lymphoma cells with RI-TATp53C′ peptide induces apoptosis. Cells were treated with wild-type or mutant peptide, and DNA content was analyzed by flow cytometry 24 h after peptide addition. (B) Long-term survival of SCID mice harboring lethal peritoneal Namalwa lymphoma tumor burden after RI-TATp53C′ peptide treatment. Namalwa lymphoma cells were IP injected into SCID mice and allowed to proliferate for 48 h. Mice were then injected 16 times over 20 d with vehicle control (*n* = 16), 900 μg of wild-type RI-TATp53C′ peptide (*n* = 12), or 900 μg of mutant peptide (*n* = 6). Mean survival duration was 35 d for vehicle-treated mice and 33 d for mice receiving mutant peptide, whereas 50% of mice treated with wild-type RI-TATp53C′ peptide remained healthy at 150 d after tumor cell injection.

To broaden these results, we also tested the efficacy of the RI-TATp53C′ peptide in a mouse model of aggressive, disseminated peritoneal lymphoma. Wild-type RI-TATp53C′ peptide, but not mutant peptide, induced G1 phase accumulation and substantial apoptosis in Namalwa human lymphoma cells (Figure 5A). When injected IP into SCID (severe combined immune deficiency) mice, Namalwa cells proliferate in the peritoneum and disseminate to other locations (e.g., spleen, lymph nodes, and blood [de Menezes et al. 1998]), modeling human B-cell lymphoma (Bertolini et al. 2000). Mice harboring peritoneal lymphoma succumbed to tumor burden with similar kinetics when treated with either vehicle or mutant peptide, with a mean survival time of 35 d and 33 d, respectively (Figure 5B). In contrast, wild-type RI-TATp53C′ peptide treatment resulted in 50% long-term survival (*p* < 0.0007) (Figure 5B), with six of 12 treated mice still healthy at more than 200 d after tumor cell injection. Taken together, these observations demonstrate that in models of terminal metastatic human disease, transducible p53-activating peptides can modulate tumor biology in vivo, resulting in significantly decreased tumor burden, increased lifespan, and long-term disease-free survival.

**Figure 6:**
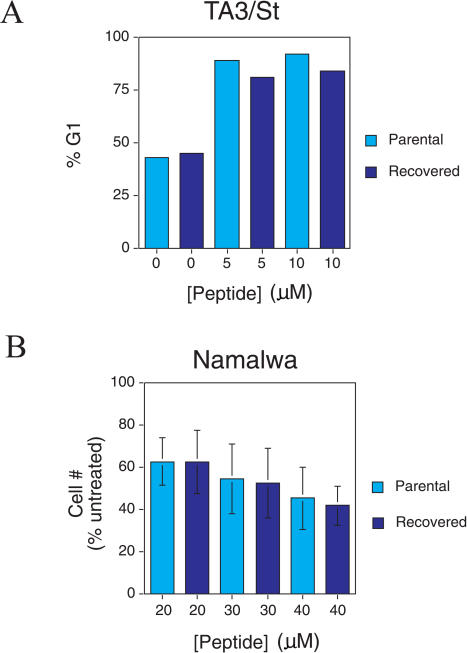
Tumor-Reconstituted Cells from Treated Mice Remain Sensitive to RI-TATp53C′ Peptide-Induced G1 Arrest or Apoptosis in Culture (A) TA3/St cells were recovered from an A/J mouse treated with RI-TATp53C′ peptide and grown in DMEM/10% FBS. Recovered cells were treated with increasing concentrations of RI-TATp53C′ peptide and then analyzed for DNA content by flow cytometry 24 h later. (B) Namalwa cells were recovered from a SCID mouse treated with RI-TATp53C′ peptide and grown in RPMI plus 10% FBS. Recovered cells were treated with increasing concentrations of RI-TATp53C′ peptide. After 2 d, the number of viable cells was assessed by Trypan blue exclusion and normalized to the number of viable untreated cells. Mean and standard deviation of multiple experiments are depicted.

The subset of RI-TATp53C′ peptide-treated animals that succumbed to peritoneal carcinomatosis could have failed treatment either because of the emergence of peptide-resistant tumor cells or because of insufficient treatment duration. To distinguish between these two possibilities, we isolated TA3/St and Namalwa cells from animals that failed treatment. In both cases, the cells readily proliferated in culture under the same conditions as the parental cell population (data not shown). RI-TATp53C′ peptide treatment of reconstituted TA3/St cells induced a G1 arrest similar in extent to that of the parental cell line (Figure 6A). RI-TATp53C′ peptide treatment of reconstituted Namalwa cells also inhibited the viability of both parental and reconstituted cells to the same degree (Figure 6B). These observations demonstrate that treatment failure is not due to acquisition of RI-TATp53C′ peptide resistance and suggests that an extended treatment protocol (greater than 12 d) may lead to a further enhancement of survival in these preclinical cancer models.

## Discussion

Advanced-stage peritoneal carcinomatosis and disseminated peritoneal lymphomas are often resistant to current chemotherapy treatment (Parsons et al. 1996), and new strategies for treating these diseases are clearly needed. The need to develop different therapeutic modalities to restore tumor suppressor function is acutely illustrated by the current limitations of viral/DNA-based strategies for delivering tumor suppressor genes to cancer cells in patients (McCormick 2001). Here we show that macromolecular biological cargo can be delivered via TAT-mediated transduction in order to modulate tumor biology in vivo. Specifically, we find that delivery of a transducible p53-activating peptide in sensitive tumor cells inhibits solid tumor growth in vivo (see Figure 3) and dramatically extends survival (greater than 6-fold), yielding disease-free animals in terminal peritoneal cancer models of human metastatic disease (see Figures 4 and 5).

The vast majority of tumors express either wild-type p53 protein or a full-length p53 point mutant (Vousden and Lu 2002). This observation has led to the hypothesis that reactivation of endogenous p53 protein will be a useful means of treating cancer. The data presented here provide evidence for this hypothesis by showing that TAT-mediated delivery of a p53-activating peptide in vivo is an effective treatment for multiple preclinical cancer models. This macromolecular approach to p53 reactivation has certain advantages over the limited number of small molecule-based strategies reported to reactivate mutant p53 in vivo (Foster et al. 1999; Bykov et al. 2002). First, the RI-TATp53C′ peptide can activate wild-type p53 in addition to several p53 contact mutants. Second, small molecules may suffer from a lack of specificity (Rippin et al. 2002) in comparison to larger, more information-rich macromolecules. Finally, recent investigations into the mechanism of TAT-mediated transduction (Richard et al. 2002; Fittipaldi et al. 2003) suggest that, unlike small molecules, TAT-linked cargo is taken up by macropinocytosis (Wadia et al. 2004) and is therefore not susceptible to the multidrug resistance phenotype. Theoretically, tumors could avoid the RI-TATp53C′ peptide action by mutating or deleting p53; however, we did not observe peptide resistance here (see Figure 6). Therefore, we conclude that linking PTDs to p53C′ and to other p53-activating peptides may be an effective therapeutic strategy applicable to a significant fraction of human cancers.

The work presented here provides several broad lines of evidence for the general feasibility of applying in vivo TAT-mediated transduction to cancer therapy. First, we find that inversion of the p53C′TAT peptide sequence and synthesis with D-amino acids results in a highly stable peptide (RI-TATp53C′) that retains both biological activity and the ability to transduce into cells. Given the rapid degradation of L-residue-containing peptides in vivo (Chorev and Goodman 1993), use of retro-inverso transformations with D-isomer residues and/or other stabilizing procedures will likely be essential for the pharmacological use of transducible peptides. Second, given the history of virus-mediated gene delivery, the necessity of validating new therapeutic approaches to systemic disease in the context of an intact immune system cannot be underestimated. Consequently, here we demonstrate that TAT-mediated systemic delivery inhibits tumor growth in immune competent animals.

Finally, most studies on anticancer transduction peptides have relied primarily on the use of solid, subcutaneous tumor growth as a measure of efficacy (Datta et al. 2001; Harada et al. 2002). Although informative, such studies are inherently limited by the minimal impact that subcutaneous tumors have on the biology of the host and by the failure of this type of tumor to closely mimic human disease. In contrast, the more rigorous peritoneal carcinomatosis and peritoneal lymphoma models used here require that therapeutic agents be able to suppress tumors to such an extent that the deleterious effects of the tumor on host physiology are substantially ameliorated. This is a particularly salient point because cancer patients generally do not succumb to the primary tumor burden but to complications from metastatic disease (Fidler 2003). Indeed, anticancer therapeutics are defined as clinically successful by their ability to alleviate pathology and extend survival and not simply by their ability to reduce tumor volume. Our work here, combined with that of Fulda et al. (2002), demonstrates that transducible agents can effectively treat rigorous models of terminal cancer.

Current clinical use of macromolecular biological therapies is limited to agents that have an extracellular mode of action. The preclinical data presented here demonstrate a proof-of-concept that intracellular delivery of biologically active macromolecular cargo by TAT-mediated transduction can modify specific pathways in vivo and that this approach potentially serves as a foundation for the generation of new classes of intracellular biological therapeutics.

## Materials and Methods

### 

#### Cell culture and flow cytometry.

TA3/St (gift of W. G. Kaelin), H1299 (gift of R. K. Brachmann), and human foreskin fibroblast (M. Haas) cells were maintained in DMEM plus 10% fetal bovine serum (FBS) and penicillin/streptomycin (P/S). Namalwa cells (American Type Culture Collection, Manassas, Virginia, United States) were maintained in RPMI plus 10% FBS, P/S. HCT116 cells (gift of B. Vogelstein) were grown in McCoy's medium plus 10% FBS, P/S. All cells were maintained at 37°C in 5% CO_2_. Short-term cell viability was assessed by counting Trypan blue-excluding cells on a hemocytometer. Long-term cell viability was assessed by colony formation assay. After serial dilution and 10 d of culture, colonies were washed in PBS and stained with 1% methylene blue. Cellular senescence was assessed by X-Gal staining as previously described (Schwarze et al. 1999), except for the use of PBS (pH 6.0). For cell cycle analysis, TA3/St cells were treated with 0.25–10 μM peptide and Namalwa cells with 40 μM peptide. DNA was stained 24 h later with 10 μg/ml propidium iodide in 0.5% NP-40 (TA3/St cells) or Draq5 (Namalwa cells) per the manufacturer's instructions (Qbiogene, Carlsbad, California, United States). DNA profiles were analyzed using a FACScan and CellQuest software (Becton Dickinson, Palo Alto, California, United States).

#### Peptide synthesis.

Peptides were synthesized by standard Fmoc chemistry on an ABI 433A Peptide Synthesizer (Applied Biosystems, Foster City, California, United States). Crude peptides were purified by reverse-phase HPLC over a C18 preparatory column (Varian, Palo Alto, California, United States). The identity of all peptides was confirmed by mass spectrometry.

#### Promoter activity assays.

In a 96-well dish, 4 × 10^4^ cells were plated per well. The next day, H1299 cells were transfected with 15 ng of TK-Renilla (Promega, Madison, Wisconsin, United States), 200 ng of PG13-Luc, and one of the following: 0.3 ng of empty vector, 0.3 ng of p53 expression vector, or 1 ng mutant of p53 expression vector (gift of R. K Brachmann). SW480 cells were transfected with 25 ng of TK-Renilla and 250 ng of PG13-Luc reporter plasmid. Cells were all transfected using Lipofectamine 2000 per the manufacturer's protocol (Invitrogen, Carlsbad, California, United States). After 5 h, the transfection medium was removed and peptides were added to cells. Luciferase activity was measured 24 h later with the Dual Luciferase Reporter Assay System per the manufacturer's instructions (Promega).

#### Animal tumor models.

For TA3/St tumor models, 4- to 8-wk-old immune competent A/J female mice were obtained from Jackson Laboratory (Bar Harbor, Maine, United States). Solid TA3/St tumors were generated by subcutaneous injection of 1.5 × 10^6^ TA3/St cells in 200 μl of Hanks' balanced salt solution (HBSS). Tumor volume was estimated by *V* = (*a* ^2^ × *b*)/2, where *a* is the short axis and *b* is the long axis of the tumor. IP TA3/St tumors were generated by injection of 2 × 10^6^ TA3/St cells IP in 400 μl of HBSS. For the Namalwa lymphoma tumor model, 6- to 8-wk-old CB17 SCID female mice were obtained from Charles River Laboratory (Wilmington, Massachusetts, United States). Then, 5 × 10^5^ Namalwa lymphoma cells were injected IP in 400 μl of HBSS. Peptide was dissolved in water, brought to 600 μl in PBS, and injected IP. All animal studies were approved by the University of California, San Diego, Institutional Animal Care and Use Committee.

#### Histology.

Mice harboring solid TA3/St tumors were injected with 650 μg of biotinylated RI-TATp53C′ peptide and sacrificed 1 h postinjection. Sections from frozen tumors were stained with Vectastain Elite ABC Kit and DAB substrate per the manufacturer's instructions (Vector Laboratories, Burlingame, California, United States).

#### Statistical analysis.

Student's *t*-test was used to determine statistical significance (*p* < 0.05) in all experiments except animal survival experiments, in which the Wilcoxon Rank-Sum Test was performed.

## Abbreviations

A/J: A/Jax strain
FBS: fetal bovine serum
HBSS: Hanks' balanced salt solution
IP: intraperitoneal
P/S: penicillin/streptomycin
PTD: protein transduction domain
SCID: severe combined immune deficiency
